# Enhanced Characterization of the Smell of Death by Comprehensive Two-Dimensional Gas Chromatography-Time-of-Flight Mass Spectrometry (GCxGC-TOFMS)

**DOI:** 10.1371/journal.pone.0039005

**Published:** 2012-06-18

**Authors:** Jessica Dekeirsschieter, Pierre-Hugues Stefanuto, Catherine Brasseur, Eric Haubruge, Jean-François Focant

**Affiliations:** 1 Department of Functional and Evolutionary Entomology, Gembloux Agro-Bio Tech, University of Liege, Gembloux, Belgium; 2 Department of Chemistry, Organic and Biological Analytical Chemistry, CART, University of Liege, Liege, Belgium; Duke University, United States of America

## Abstract

Soon after death, the decay process of mammalian soft tissues begins and leads to the release of cadaveric volatile compounds in the surrounding environment. The study of postmortem decomposition products is an emerging field of study in forensic science. However, a better knowledge of the smell of death and its volatile constituents may have many applications in forensic sciences. Domestic pigs are the most widely used human body analogues in forensic experiments, mainly due to ethical restrictions. Indeed, decomposition trials on human corpses are restricted in many countries worldwide. This article reports on the use of comprehensive two-dimensional gas chromatography coupled with time-of-flight mass spectrometry (GCxGC-TOFMS) for thanatochemistry applications. A total of 832 VOCs released by a decaying pig carcass in terrestrial ecosystem, *i.e*. a forest biotope, were identified by GCxGC-TOFMS. These postmortem compounds belong to many kinds of chemical class, mainly oxygen compounds (alcohols, acids, ketones, aldehydes, esters), sulfur and nitrogen compounds, aromatic compounds such as phenolic molecules and hydrocarbons. The use of GCxGC-TOFMS in study of postmortem volatile compounds instead of conventional GC-MS was successful.

## Introduction

The decay process of vertebrates begins rapidly after death (*i.e.* four minutes after death) [1] and leads to the release of postmortem compounds in the ecosystem [2]–[3]. These cadaveric compounds, mainly volatile organic compounds (*i.e.* VOCs), are by- or end-products of the decay process [2], [4]. They come from the catabolism of the four major categories of biological macromolecules in living organisms: proteins, nucleic acid, lipids and carbohydrates [2]–[3]. The principal decay pathways and the metabolic origin of the main vertebrate postmortem volatiles were reviewed by Dent and colleagues [5], Boumba and colleagues [6] and recently by Paczkoski and Schütz [4]. However, the metabolic origin of many cadaveric compounds is still unknown [4], [7]. Only a few research groups have studied the postmortem VOCs emanating from human remains [8]–[13] and animal carcasses (pig [7], [14]–[15], mouse [16], rabbit [17]). Nevertheless, the majority of these studies is focused on burial decomposition or in closed environments (“body bag”) and limits the access to the corpse for the necrofauna, mainly insects [7]–[11]. The available information concerning postmortem chemistry of above-ground decomposition is rather limited. Numerous applications would however benefit from a better understanding of the postmortem volatiles emitted during the decay process.

The cadaveric VOCs find applications in forensic sciences and the etiology of death [3], in training of cadaver dogs (human remains detection or HRD detection) [12]–[13], [18]–[20], in the development of cadaveric material detection devices [8]–[10], [17] (electrochemical sensors (“electronic nose”) [8]–[9], [17], [21], in exploiting insect olfaction (biosensor or biodetector [4], [22]–[23]) or in the determination of the post-mortem interval (PMI) [1], [3]–[4]. The smell of death is constituted by a blend of chemical compounds changing over time and according to animal remains [8]–[9], [14]. Indeed, a recent study has shown that the odor of human remains is different from that of animals [13].

For the analysis of cadaveric VOCs, gas chromatography (GC) coupled to mass spectrometry (MS) is the technique of choice [3], [7]–[13], [24]. However, the volatile profile of the decomposition odor is constituted by a large number of VOCs [8]–[9] and GC easily suffers from peak capacity limitations. The complexity of volatile postmortem samples requires the use of more complex analytical methods. For example, GC is unable to detect quantitatively both short and long chain acids (*e.g*. present in decomposition fluid) due to the polarity difference in the molecules [25]–[26]. Comprehensive two-dimensional gas chromatography (GCxGC) has been developed to meet an increasing need for complex sample analysis and to address limitations such as peak capacity, dynamic range and restricted specificity of one-dimensional (conventional) GC systems (1D-GC) (*i.e.* to improve the global efficiency of the separation). GCxGC can be defined as a chromatographic technique during which a sample is subjected to two different separation processes coupled online [27] and as a result every compound is characterized by a retention time in each dimension (^1^t_R_ and ^1^t_R_) [4]. In practice, the end of the first dimension (^1^D) column is placed in a temperature controlled interface named ‘the modulator’ and further serially connected to the second dimension (^2^D) column. The cryogenic modulator ensures high sampling rates and transfer of the sample to ^2^D column [28]. The entire ^1^D chromatogram is thus ‘sliced’ following a modulation period (P_M_) of a few seconds and sent into ^2^D for a fast GC-type separation, resulting in peak widths of 200–600 ms [29]. By fine-tuning of the GC phase combination, compounds potentially still coeluting at the end of the ^1^D separation can be separated on the basis of their different behavior as regards of the ^2^D phase. Globally, the separation power is increased and the sensitivity is also enhanced by cryogenic zone compression [30], [31]. In terms of detector, in addition to flame-ionization and other element selective detectors, various mass-spectrometric (MS) detectors, providing structural information (an additional dimension), can be used since the first coupling was reported in early 2000 [32]. Although double focusing sector, quadrupole, and ion traps are popular MS detectors for GC, they have limited use in GCxGC because of their relatively slow scanning rates, compared to fast acquisition time-of-flight (TOF) MS better suited to characterize very narrow ^2^D peaks. Additionally, the absence of concentration skewing in the TOFMS instrument ensures spectral continuity and allows mass spectral deconvolution of coeluting chromatographic peaks characterized by different fragmentation patterns [33]. This capability allows the identification of different compounds if the peak apexes of coeluting analytes are at least separated by three scans and differ somewhat in their mass spectra. The use of deconvoluted ion current (DIC) makes the TOFMS almost like a third dimension for the separation system. Consequently, the GC×GC–TOFMS coupling is a powerful instrument combining improved chromatographic resolution of the GC × GC and the analytical resolving power of the TOFMS [34]–[35]. GCxGC-TOFMS, has thus been used to analyze complex samples in various fields [36], including VOC analyses [37]–[39].

**Table 1:** The five decompositional stages defined in this study and their descriptions.

Decompositional stage	Description	Literature report
(1) *Fresh*	From death until the first signs of bloating	[1], [49], [52], [54]
	Autolysis	
(2) *Bloated*	Putrefaction mechanism generates accumulation of breakdown gases causingbloating of the corpse. The first signs of the bloated stage appear in the abdomen.Then the whole body swells	[1], [52], [54], [56]
	Anaerobic fermentations	
(3) *Active decay*	Darkening of the skin	[1], [52], [54], [56]
	The skin is breaking up and the body began to deflate. Protein sources arebroken down into fatty acids and other decomposition products such as skatole,indole, cadaverine, putrescine	
(4) *Advanced decay*	Corpse dries and the remains are skin, cartilage, hair, bones and some fragmentsof flesh	[52], [54], [56]
(5) *Dry remains or skeletonisation*	The only remains are bones and hair	[52], [54]
	Diagenesis	

**Figure 1:**
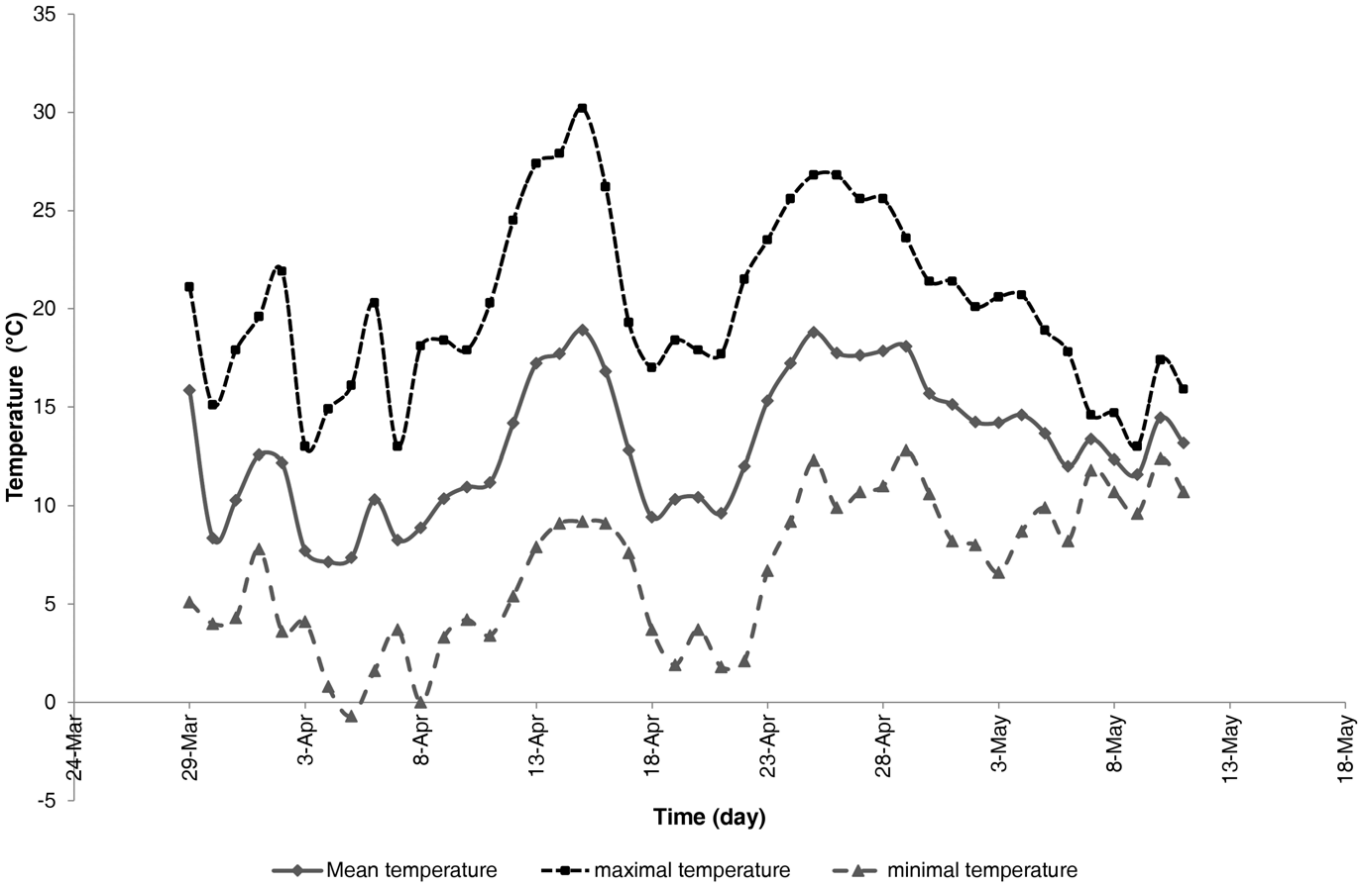
Temperature recordings in the forest biotope on the lateral cage of the pig carcass.

**Figure 2:**
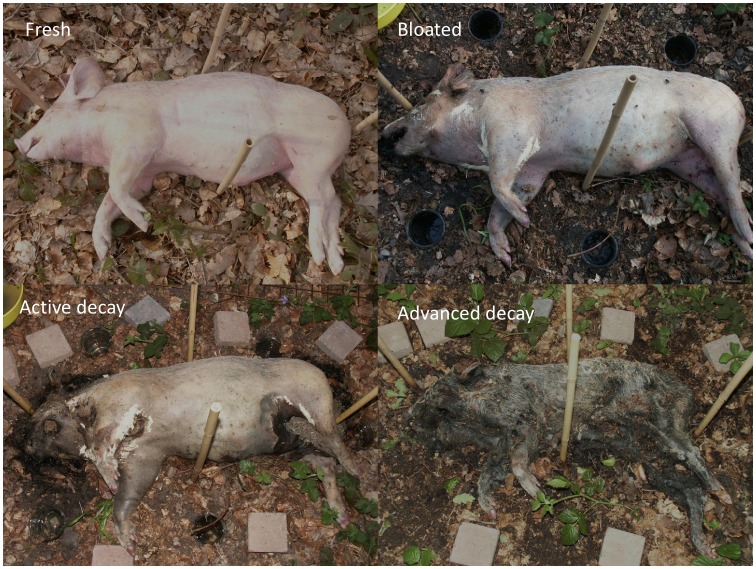
Typical decay stages followed by the pig carcass in a forest biotope.

**Figure 3:**
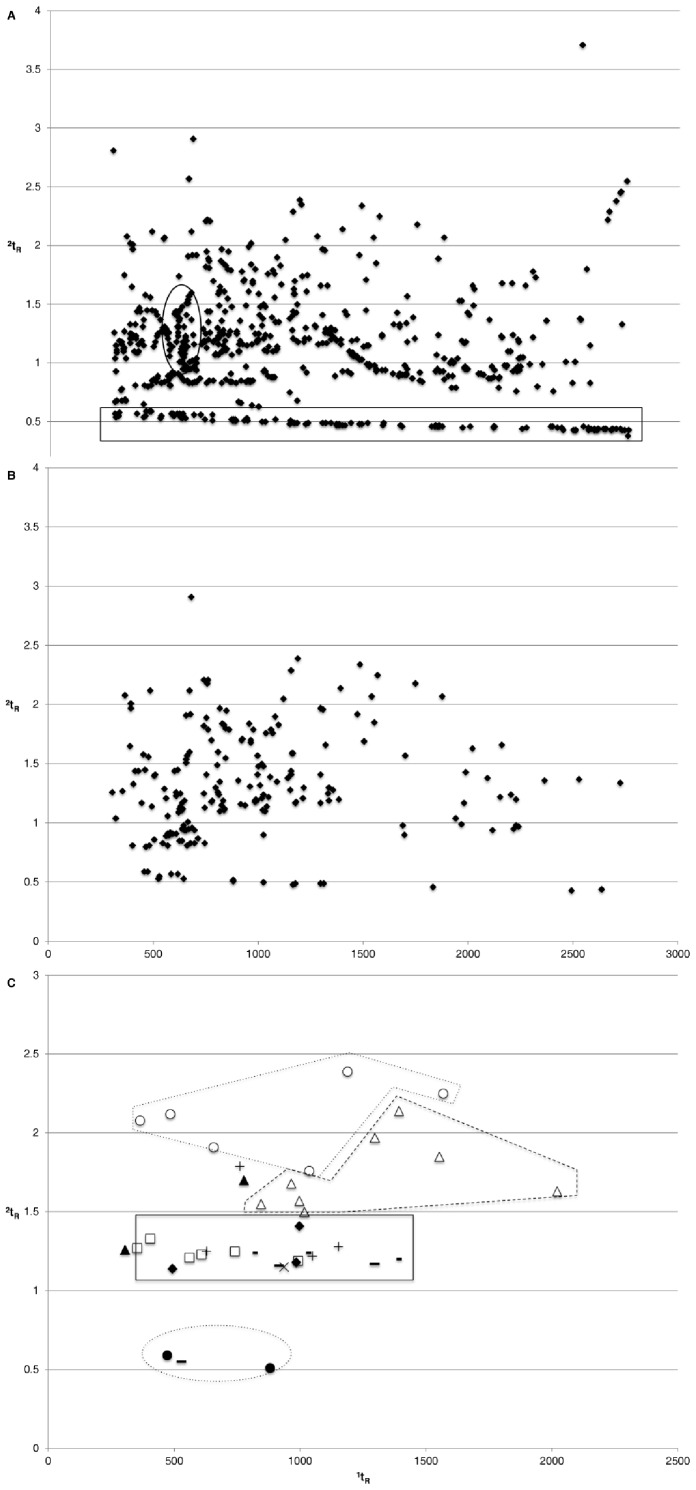
GCxGC-TOFMS apex plot of a sample (1^st^ of May) of the advanced decay stage. All t_R_ are in seconds. (A) 633 hits were identified after raw data processing. (B) 218 peaks were identified after removal of column bleed (rectangle region), solvent signals (circled region), and analytes present in the reference blank samples. (C) chromatographic distribution of the 42 specific VOC compounds present in that particular sample and in at least three other samples (♦Alcohols; + Aldehydes; ○ Amides; • Amines; △ Aromatic compounds; Υ Carboxylic acids; × Ester; – Ketones; ▴ Sulfur compounds; − Others compounds).

**Table 2:** List of occurrences for the detected postmortem chemical compounds by GCxGC-TOFMS.

8 occurrences	7 occurrences	6 occurrences	5 occurrences	4 occurrences
✓1H-indole	✓1-Pentene, 2-methyl-	✓1-Butanol	✓1-Octanol	✓1-Hexen-3-ol
	✓1,2,3-Propanetriol	✓Butanoic acid	✓1-Propanol, 2-methyl-	✓1-Pentanol, 4-methyl-
	✓Ethanol	✓Propanoic acid, 2-methyl-	✓Butanoic acid, 2-methyl	✓2-Hexanol
	✓Phenol, 4-methyl-	✓Disulfide, dimethyl	✓Butanoic acid, 3-methyl	✓2-Hexen-1-ol
	✓Acetaldehyde	✓Trisulfide, dimethyl	✓Pentanoic acid	✓Hexanoic acid
		✓1-Butanamine, 3-methyl-	✓Benzenemethanol, à-methyl-	✓Pentanoic acid, 4-methyl-
		✓2-Propanol, 1-amino-	✓Pyrazine, tetramethyl-	✓Naphthalene, 2,6-diisopropyl
		✓Formamide, N-butyl-	✓Pyrazine, trimethyl-	✓Quinazoline, 2,4-dimethyl-
		✓Formamide, N,N-dimethyl-	✓1-Butanol, 4-Amino-	✓Quinoline
		✓1,3-Dioxolane, 2-acetyl-	✓Formamide, N-methyl-	✓Acetic acid, ethyl ester
			✓Trimethylamine	✓Butanoic acid, 3-methyl-, butyl ester
			✓Nonanal	✓Dothiepin*
			✓2-Octanone	✓2-Piperidinone
			✓2-Undecanone	✓Acetamide, N-methyl-
			✓Butyl isocyanatoacetate	✓Butanamide
			✓Hydroperoxide, 1-ethylbutyl	✓Formamide, N-phenyl-
				✓Hexanamide, N-methyl
				✓Methanamine, N,N-dimethyl-
				✓Benzaldehyde
				✓Heptanal
				✓Nonenal
				✓Propanal, 2,2-dimethyl-
				✓2-Nonanone
				✓3-Octanone
				✓Cyclohexanone
				✓Ethanone, 1-phenyl-
				✓Propane, 1-bromo-2-methyl-
				✓Hydroperoxide, 1-methylbutyl
**1 compound**	**5 compounds**	**10 compounds**	**16 compounds**	**28 compounds**

•Dothiepin is not considered as a cadaveric compound.

Here, we report on a first field trial using GCxGC-TOFMS for the study of postmortem VOCs released by above-ground decomposition of pig carcasses, using it as a human body analogue.

## Materials and Methods

### 1. Animal Model and Field Site

Domestic pig (*Sus scrofa domesticus* L.) (25 kg) was used to surrogate human models mainly for physiological, biochemical, ethical, legal and economic reasons [25], [40]–[45]. Unlike other animals, pigs are considered to be an acceptable substitute due to their similarity to humans in body mass (torso in weight), skin structure, fat to muscle ratio and hair coverage [5], [25], [45]–[46]. The greatest dissimilarity between pigs and humans are the bones, which have a different microstructure [46]–[47]. The piglet was killed by a penetrative captive bolt and disposed in the experimental site within the next 4 hours. Immediately after the euthanasia, the pig carcass was packed in a double plastic bag to avoid any insect colonization before laying on the experimental biotope. This study was approved by the committee on the Ethics of Animal Experiments of the University Faculty of Agricultural Sciences of Gembloux (since 2009, Gembloux Agro-Bio Tech, University of Liege).

The study site was a forest biotope, located in Belgium (Lambert-coordinates: 141512.00/149844.00) with pedunculate oaks (*Quercus robur* L.), European beeches (*Fagus sylvatica* L.) and sycamore maples (*Acer pseudoplatanus* L.). This field study was conducted with the permission of the forest administrator.

The decaying pig was placed in metal mesh cages (180 cm×90 cm×90 cm) to avoid scavenging by carnivores. The experiment was conducted during six weeks in spring 2007 (March 29-May 11). As control samples, volatile collection of the atmospheric VOCs was performed simultaneously to the pig samples, at 50 m from the decomposing swine carcass.

As temperature is one of the most important parameters influencing the decomposition rate [1]–[2], [48]–[50], the ambient air temperature was automatically measured once an hour using a data logger (Testo 175-T1® temperature data logger, Germany) placed on the lateral side of the cage, at a height of 75 cm. The daily mean temperature was calculated on the basis of ambient air temperature recorded at a time interval of 24 hours. Other environmental parameters (humidity, wind velocity, wind direction) were recorded thanks to Vantage Pro Plus™ Stations® (Davis, Hayward, CA, USA).

**Figure 4:**
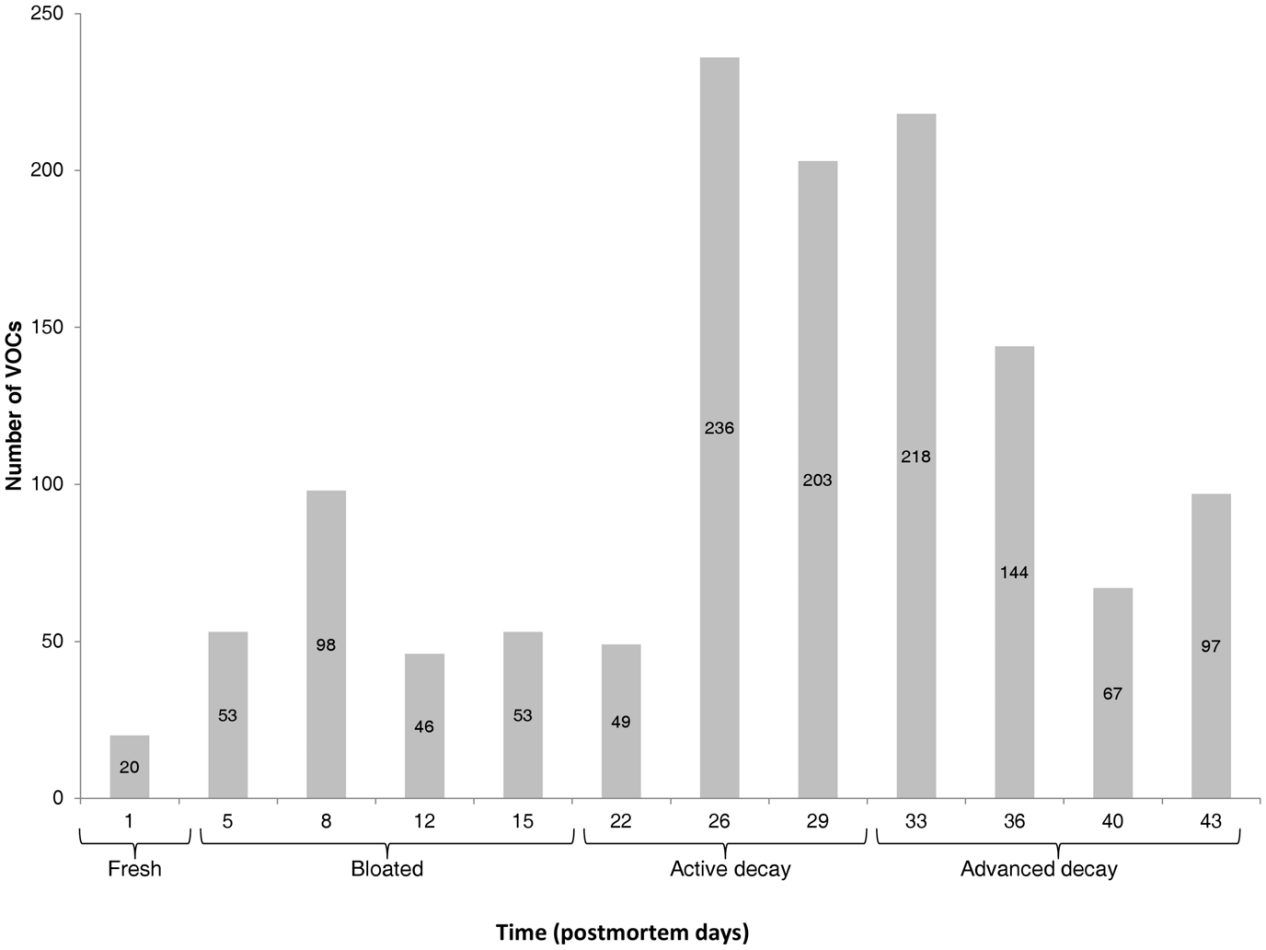
Number of released compounds according to the decay stages and postmortem time.

**Figure 5:**
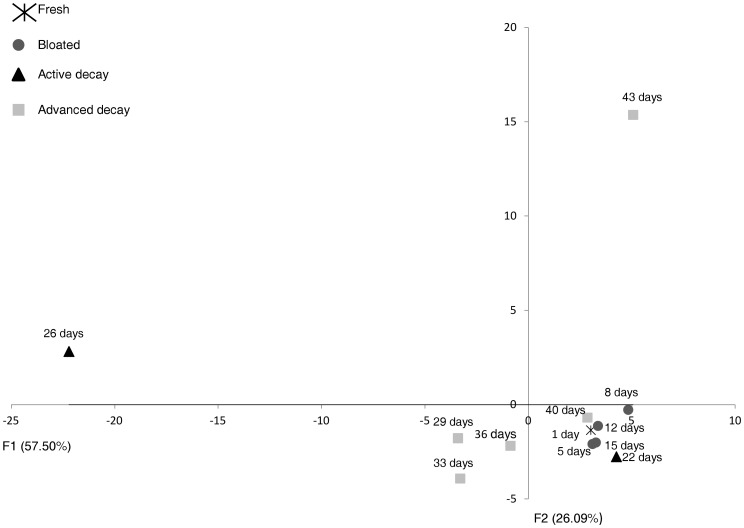
Spatial distribution of Y-variables in a score-plot based on relative area of VOCs.

**Figure 6:**
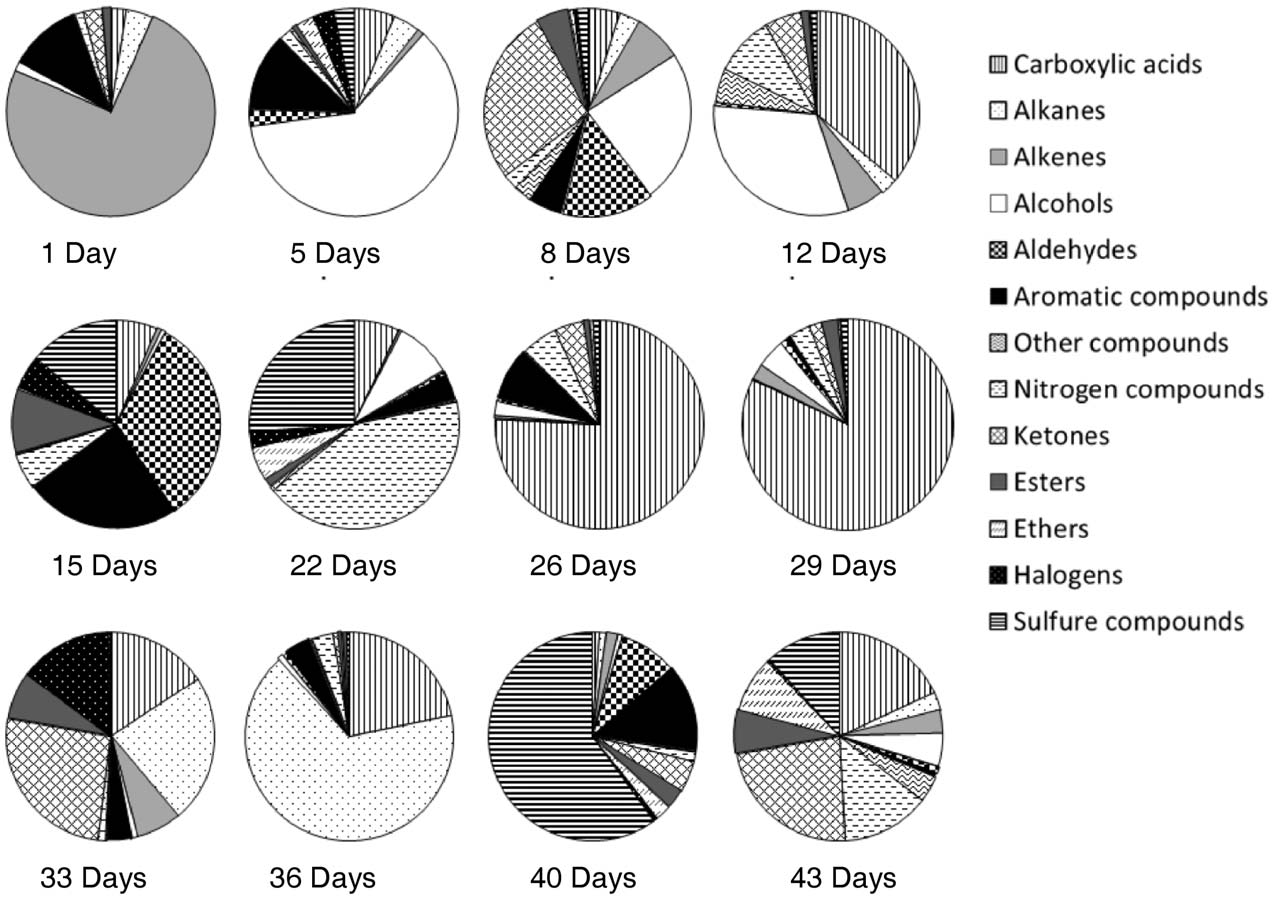
Distribution of chemical classes according to postmortem time (days).

**Table 3:** Volatile chemicals released in the headspace of decaying pig carcass according to the decay stages, ordered by chemical families.

VOCs	Decay stages				Literature report
	Fresh	Bloated	Active decay	Advanced decay	
**Alkanes**					
Butane	▴	–	–	▴	[17]
Cyclopentane, butyl-	–	–	▴	▴	N.R.
Dodecane	–	▴	▴	–	[7]
Eicosane	–	▴	–	▴	[14]
Heneicosane	▴	–	–	–	N.R.
Heptacosane	–	▴	▴	–	N.R.
Heptane, 2,4-dimethyl-	–	–	–	▴	[3]
Hexadecane	–	▴	▴	▴	[59]
Nonane	–	▴	–	▴	[7]
Octane	–	▴	–	▴	[3], [10]–[11], [17]
Tetradecane	–	▴	▴	▴	[7], [59]
Tricosane	–	–	▴	▴	N.R.
Tridecane, 4-methyl-	–	▴	▴	–	N.R.
Undecane	–	▴	–	▴	[7]–[9]
Undecane, 2,5-dimethyl-	–	▴	–	–	N.R.
**Alkenes**					
1-Butene, 3-methyl-	–	–	▴	▴	N.R.
1-Decene	–	–	▴	–	N.R.
1-Pentene, 2-methyl-	–	▴	▴	▴	N.R.
1,3,6-Octatriene, 3,7-dimethyl-	–	–	▴	–	N.R.
α-Phellandrene	–	▴	▴	–	N.R.
α-Pinene	–	–	▴	▴	[3], [7], [15]
γ-Terpinene	▴	–	▴	▴	N.R.
α-Thujene	–	▴	▴	–	N.R.
o-Xylene	▴	▴	–	–	[3], [7], [9], [11], [13]
p-Xylene	–	–	–	▴	[3], [8]–[13]
**Alcohols**					
1-Butanol	–	▴	–	▴	[3]–[5], [7], [13]–[14], [17]
1-Heptanol	–	–	▴	▴	[15]
1-Hexanol	–	▴	▴	–	[3]–[4], [7], [12]–[13], [15]
1-Hexen-3-ol	–	▴	▴	–	N.R.
1-Octanol	–		▴	▴	[4], [12]–[13], [15]
1-Pentanol	–	–	▴	▴	[3]–[4], [6], [9], [12]–[14], [17]
1-Pentanol, 4-methyl-	–	–	▴	▴	N.R.
1-Propanol, 2-methyl-	–	▴	▴	▴	[3]–[4], [6]–[7], [14]
1-Tridecanol	–	▴	–	–	N.R.
1,2,3-Propanetriol	–	▴	▴	▴	N.R.
2-Hexanol	–	▴	▴	▴	N.R.
2-Hexen-1-ol	–	▴	▴	–	N.R.
3-Hexanol	–	▴	–	▴	N.R.
3-Hexene-2,5-diol	–	▴	–	▴	N.R.
7-Octen-4-ol	–	–	–	▴	N.R.
Cyclohexanol	–	▴	▴	–	N.R.
Cyclopentanedecaol	–	▴	▴	–	N.R.
Ethanol	▴	▴	▴	▴	[3]–[6], [11], [13]–[15]
**carboxylic acids**					
2,3-Dihydroxysuccinic acid	–	▴	–	▴	N.R.
3-Pentenoic acid, 4-methyl-	–	–	▴	▴	N.R.
Butanoic acid	–	▴	▴	▴	[1], [3]–[5], [12]–[14], [17], [25], [49]
Butanoic acid, 2-methyl	–	–	▴	▴	[3], [9], [14], [17]
Butanoic acid, 3-methyl- (iso-valeric)	–	▴	▴	▴	[14], [25]
Butyric acid, γ-amino-	–	–	▴	▴	N.R.
Heptanoic acid	▴	–	▴	▴	N.R.
Hexadecanoic acid (palimitic)	–	–	▴	▴	[2], [4], [25]
Hexanoic acid (caproic)	–	▴	▴	▴	[4], [12]–[14]
Isobutanoic acid, α-Amino	–	–	▴	–	N.R.
Octanoic acid (caprylic)	–	–	▴	▴	[2], [13]–[14]
Pentanoic acid (valeric)	–	–	▴	▴	[12]–[14], [25]
Pentanoic acid, 4-methyl- (iso-caproic)	–	–	▴	▴	[14], [25]
Propanoic acid, 2-methyl- (iso-butyric)	–	–	▴	▴	[14], [25]
Propanoic acid, 2,2-dimethyl-	–	–	▴	▴	N.R.
succinic acid, 2,3-dihydroxy-	–	–	▴	▴	N.R.
**Aromatic coumpounds**					
1(3H)-Isobenzofuranone	–	–	▴	▴	N.R.
1H-Indole	–	▴	▴	▴	[3]–[5], [12]–[15]
1H-Indole, 3-methyl-	–	–	▴	▴	N.R.
1H-Pyrrole, 2,5-dimethyl-	–	–	▴	▴	N.R.
Benzene, 1-methyl-4-(1-methylethyl)-	–	–	▴	▴	[14]
Benzene, 1-methylethyl-	–	▴	–	▴	N.R.
Benzene, ethyl-	–	▴	–	▴	N.R.
Benzene, methyl-	–	▴	▴	–	N.R.
Benzeneethanol	–	–	▴	▴	N.R.
Benzenemethanol	–	–	▴	–	N.R.
Benzenemethanol, α-methyl-	–	–	▴	▴	N.R.
Furan, 2-pentyl-	–	–	▴	▴	[12]–[13]
Isoquinoline	–	–	–	▴	N.R.
Naphthalene	–	–	▴	▴	[4], [7]–[9], [11], [13]
Naphthalene, 2,6-diisopropyl	–	–	▴	▴	N.R.
Phenol, 2-ethyl-	–	–	▴	▴	N.R.
Phenol, 4-ethyl-	–	–	▴	–	[15]
Phenol, 4-methyl-	–	–	▴	▴	[4], [11], [13]–[15]
2-phenylethanol	–	–	▴	▴	[14]
Pyrazine, 2-butyl-3,5-dimethyl	–	–	▴	▴	N.R.
Pyrazine, 2,3-dimethyl-	–	–	▴	▴	N.R.
Pyrazine, 2,5-dimethyl-	–	–	▴	▴	N.R.
Pyrazine, 2,6-dimethyl-	–	–	▴	▴	N.R.
Pyrazine, 3-ethyl-2,5-dimethyl-	–	–	–	▴	N.R.
Pyrazine, 3,5-diethyl-2-methyl-	–	–	–	▴	N.R.
Pyrazine, methyl-	–	–	▴	▴	N.R.
Pyrazine, tetramethyl-	–	–	▴	▴	N.R.
Pyrazine, trimethyl-	–	–	▴	▴	[14]
Pyridine, 2-methyl-	–	–	▴	▴	N.R.
Pyridine, 2,6-dimethyl-	–	–	▴	▴	N.R.
Quinazoline	–	–	▴	–	[14]
Quinazoline, 2,4-dimethyl-	–	–	▴	▴	N.R.
Quinazoline, 4-methyl-	–	–	▴	▴	N.R.
Quinoline	–	–	▴	▴	N.R.
**Esters**					
1,2-Benzenedicarboxylic acid, dihexyl ester	–	▴	–	▴	N.R.
2-Propenoic acid, 3-methoxybutyl ester	–	–	▴	–	N.R.
3-Hexen-1-ol, acetate	–	–	▴	▴	N.R.
3-octanyl acetate	–	▴	▴	–	N.R.
Acetic acid, butoxyhydroxy-, butyl ester	–	–	▴	▴	N.R.
Acetic acid, ethyl ester	–	▴	▴	▴	[3], [7], [17]
Allyl tert-Butyl carbonate	–	▴	–	▴	N.R.
Butanoic acid, 1-methylpropyl ester	–	–	▴	▴	N.R.
Butanoic acid, 3-methyl-, butyl ester	–	–	▴	▴	N.R.
Butanoic acid, butyl ester	–	–	▴	▴	[4], [9], [12], [14], [17]
Butyl 2-methylbutanoate	–	–	▴	–	[14]
Ethyl Acetate	–	▴	–	▴	[13]
Formic acid, ethenyl ester	–	▴	–	▴	N.R.
Hexadecanoic acid, ethyl ester	–	▴	–	–	N.R.
Hexanoic acid, butyl ester	–	–	▴	–	N.R.
Oxalic acid, hexyl propyl ester	–	–	▴	▴	N.R.
Propanoate, 2-hexen-1-ol	–	▴	▴	–	N.R.
Propanoic acid, 2-hydroxy-2-methyl-, ethyl ester	–	–	–	▴	N.R.
Propanoic acid, 2-hydroxy-2-methyl-, methyl ester	▴	–	▴	–	N.R.
Propanoic acid, 2-methyl-, butyl ester	–	–	▴	▴	N.R.
Propanoic acid, butyl ester	–	–	▴	▴	N.R.
Vinyl butyrate	–	▴	▴	–	N.R.
**Sulfur compounds**					
Benzenesulfonic acid, 4-hydroxy-	–	–	▴	▴	N.R.
Dicyclohexyldisulphide	–	▴	–	▴	N.R.
Disulfide, dimethyl	–	–	▴	▴	[3]–[5], [7]–[17]
Dothiepin	–	▴	▴	▴	N.R.
Methane, sulfonylbis-	–	–	▴	▴	N.R.
Sulfone, butyl isopropyl	–	▴	–	▴	N.R.
Sulfurous acid, dicyclohexyl ester	–	▴	–	▴	N.R.
Trisulfide, dimethyl	–	▴	▴	▴	[3]–[5], [7]–[11], [13]–[16]
**Nitrogen compounds**					
1-Butanamine, 3-methyl-	–	▴	▴	▴	N.R.
1-Butanol, 4-Amino-	▴	▴	▴	–	N.R.
1-Decanamine	–	▴	▴	▴	N.R.
1-Heptadecanamine	–	▴	▴	–	N.R.
2-Piperidinone	–	–	▴	▴	[14]–[15]
2-Propanol, 1-amino-	–	▴	▴	▴	N.R.
2,3-Butanediol, dinitrate	–	▴	–	–	N.R.
2,3-Dihydrooxazole, 2-t-butyl-4-(1-hydroxy-1-methylethyl)-3-methoxycarbonyl-5-methyl-	–	–	▴	▴	N.R.
5,5-Dimethylimidazolidin-2,4-diimine	▴	–	▴	–	N.R.
Acetamide	–	–	▴	–	[14]
Acetamide, N-methyl-	–	–	▴	▴	[14]
Acetamide, N,N-dimethyl-	–	–	▴	▴	[9], [14]
Acetic acid, [(aminocarbonyl)amino]oxo-	–	▴	–	▴	N.R.
Benzaldehyde, 2-amino-	–	–	▴	▴	N.R.
Butanamide	–	–	▴	▴	[14]
Butanamide, 3-methyl-	–	–	▴	▴	[14]
Formamide, (2-acetylphenyl)-	–	–	▴	▴	N.R.
Formamide, N-(2-methylpropyl)-	–	–	▴	▴	N.R.
Formamide, N-butyl-	–	–	▴	▴	N.R.
Formamide, N-methyl-	–	–	▴	▴	N.R.
Formamide, N-phenyl-	–	▴	▴	▴	N.R.
Formamide, N,N-dimethyl-	–	▴	▴	▴	[7], [14]
Heptanonitrile	–	–	▴	▴	N.R.
Hexanamide	–	–	▴	▴	N.R.
Hexanamide, N-methyl	–	–	▴	▴	N.R.
Methanamine, N,N-dimethyl-	–	▴	▴	–	N.R.
Methanediamine, N,N,N',N'-tetramethyl-	–	▴	▴	–	[13]
N-Methylvaleramide	–	–	▴	▴	N.R.
Pentanamide	–	–	▴	▴	N.R.
Propanamide	–	–	▴	–	[14]
Propanamide, 2-methyl-	–	–	▴	–	N.R.
Propanamide, N-methyl-	–	–	▴	–	[14]
Propanamide, N,2-dimethyl-	–	–	▴	▴	N.R.
Propanenitrile, 3-dimethylamino-	–	–	▴	▴	N.R.
Propylamine	–	–	▴	–	N.R.
Propylamine, N,N,2,2-tetramethyl-, N-oxide	–	–	▴	▴	N.R.
Trimethylamine	–	▴	▴	▴	[4], [7], [13]–[14]
**Aldehydes**					
2-Butenal, 3-methyl-	–	▴	▴	▴	N.R.
2-Octenal	–	–	▴	–	[12]–[13]
Acetaldehyde	–	▴	▴	▴	[3], [17]
Benzaldehyde	–	–	▴	▴	[3]–[4], [7]–[8], [12]–[14]
Heptanal	–	▴	▴	▴	[2], [4], [9], [12]–[14], [59]
Hexanal	–	▴	–	▴	[3]–[4], [10], [12]–[13]
Methylglyoxal	–	▴	▴	–	N.R.
Nonanal	–	–	▴	▴	[4], [8]–[9], [12]–[13], [17], [59]
Nonenal	–	–	▴	▴	N.R.
Octanal	–	▴	▴	–	[12]–[13]
Pentanal, 2-methyl-	–	–	▴	▴	N.R.
Propanal	–	▴	▴	–	N.R.
Propanal, 2-hydroxy-	–	▴	–	▴	N.R.
Propanal, 2,2-dimethyl-	–	▴	–	▴	N.R.
**Ketones**					
1-Octen-3-one	–	–	–	▴	N.R.
2-Cyclohexen-1-one, 3-methyl-	–	–	▴	▴	N.R.
2-Decanone	–	–	▴	▴	[15]
2-Heptanone	–	–	▴	▴	[3]–[4], [12]–[14]
2-Hexanone	–	–	▴	▴	[3], [10]
2-Nonanone	–	–	▴	▴	[1], [4], [9], [11], [13]–[15]
2-Octanone	–	–	▴	▴	[15]
2-Propanone, 1-phenyl-	–	–	▴	▴	N.R.
2-Undecanone	–	–	▴	▴	N.R.
2,3-Octadione	–	–	▴	▴	N.R.
2,4,6-Cycloheptatrien-1-one	–	–		▴	N.R.
2,5-Cyclohexadiene-1,4-dione	–	–	▴	▴	N.R.
2,5-Hexanedione	–	–	▴	▴	N.R.
2(3H)-Furanone, 5-butyldihydro- 2	–	–	▴	▴	N.R.
2(3H)-Furanone, dihydro-5-methyl-	–	–	▴	▴	N.R.
3-Hexanone	–	–	▴	▴	N.R.
3-Hexanone, 2-hydroxy-	–	–	▴	▴	N.R.
3-Octanone	–	–	▴	▴	N.R.
3-Pentanone, 2-hydroxy	–	–	▴	▴	N.R.
3-Penten-2-one, 4-methyl-	–	▴	▴	–	N.R.
4-Penten-2-one, 4-methyl-	–	▴	▴	–	N.R.
5-Hepten-2-one, 6-methyl	–	–	▴	▴	[13]
Ç-Valerolactone	–	–	▴	▴	N.R.
Cyclohept-4-enone	–	–	▴	▴	N.R.
Cyclohexanone	▴	▴	–	▴	[3]–[4], [7], [12]–[13]
Cyclopentanone	–	–	–	▴	N.R.
Cyclopentanone, 2-(1-methylpropyl)-	–	▴	▴	–	N.R.
Ethanone, 1-phenyl-	–	–	▴	▴	[1], [11], [13]–[14]
exo-5-Methyl-2-oxabicyclo[4.1.0]heptan-3-one	–	▴	▴	–	N.R.
Tridecan-2-one, 10-Methyl	–	–	▴	▴	N.R.
**Ethers**					
1,3-Dioxolane, 2-acetyl-	–	▴	▴	▴	N.R.
2-Propanol, 1-propoxy-	–	–	▴	▴	N.R.
Ethene, methoxy-	–	▴	▴	–	N.R.
Furan, 2-butyltetrahydro-	–	▴	▴	▴	N.R.
Furan, 2,3-dihydro-2,5-dimethyl-	–	–	▴	▴	N.R.
Oxirane, 2,3-dimethyl-	–	–	▴	–	N.R.
Oxiranemethanol	–	–	▴	–	N.R.
halogen compounds					
1-Chloroheptylacetate	–	–	▴	▴	N.R.
1-Iodo-2-methylundecane	–	▴	▴	–	N.R.
Acetamidine, hydrochloride-	–	▴	▴	–	N.R.
Butane, 1-bromo-2-methyl-	–	–	▴	▴	N.R.
Propane, 1-bromo-2-methyl-	–	▴	–	▴	N.R.
**Other compounds**					
Butyl isocyanatoacetate	–	▴	–	▴	N.R.
Cyanic acid, 2-methylpropyl ester	–	–	▴	▴	N.R.
Cyanic acid, propyl ester	–	▴	–	▴	N.R.
Hydroperoxide, 1-ethylbutyl	–	▴	▴	▴	N.R.
Hydroperoxide, 1-methylbutyl	–	▴	▴	▴	N.R.
Hydroperoxide, 1-methylpentyl	–	–	▴	▴	N.R.

(▴) indicated a VOC detected or (-) for a VOC not detected. The column “literature report” lists the VOCs referenced in peer-reviewed literature (number correspond to the different papers concerning the decompositional chemistry and are listed in “References” section or not referenced (N.R.).

### 2. Volatile Collection

A dynamic sampling technique was used to collect volatile organic compounds released by the decaying pig carcass. The VOCs were collected in the headspace of the decaying pig with a pump device for 1 hour at 1 Lmin^−1^ every two days during the field experiment. Simultaneously to the cadaveric VOC collection, air samples were collected as blank references. VOCs were trapped on cartridges, constituted of glass and Teflon®, containing a 40 µg SuperQ® adsorbent filter (80–100 mesh, Alltech Associates, Inc. Deerfield, IL, USA). The sorbent cartridge was connected to the pump device with Teflon® tubing and a glass funnel. The air sampling device (funnel and the sorbent cartridge) was disposed close to the abdominal cavity of the carcass (±3 cm).In the laboratory, VOCs were solvent eluted from the SuperQ® adsorbent with 150 µl of diethyl ether (HPLC grade, Sigma-Aldrich SA, Bornem, Belgium) and capped in GC-type vials. Before chromatographic analyses, liquid volatile samples were conserved at −80°C.

### 3. Chemicals

All solvents were Pestanal reagents (Riedel-de Haën, Seelze, Germany). Liquid nitrogen was purchased from Air Liquide (Liege, Belgium). Chromatographic pure grade helium gas, 99.9999%, was purchased from Air Products (Vilvoorde, Belgium).

### 4. Measurement by GCxGC-TOFMS

The GCxGC-TOFMS instrument was the Pegasus 4D (LECO Corp., St Joseph, MI, USA). This system is based on a non-moving quad-jet modulator consisting of two permanent cold nitrogen jets and two pulsed hot-air jets, which are responsible for the trapping and refocusing of compounds eluting from the first dimension (^1^D) column. This modulator was mounted in an Agilent 7890 GC oven and liquid nitrogen was used to create the cold jets. The column set was made of a 30 m Rtx®-5 (0.18 mm ID×0.20 µm df) (Restek Corp., Bellefonte, PA, USA) in the first dimension (^1^D) and a 1.0 m Rxi®-17 (0.10 mm ID×0.08 µm df) (SGE, Austin, TX, USA) in the second dimension (^2^D). The two columns were connected using a universal glass press tight connector (Restek Corp.). The modulation period (P_M_) was 4 s. The hot pulse duration was 600 ms. Helium was used as the carrier gas at a constant flow rate of 1 ml/min. 1 µl of the final extract in diethyl ether was injected into a split/splitless injector held at 200°C in splitless mode and equipped with a Double Gooseneck Restek liner. The primary oven was programmed as follows: 40°C for 5 min, at 5°C/min to 220°C and held for 5 min. The secondary oven temperature offset was 5°C, with a parallel temperature program. The modulator temperature offset was 10°C. The MS transfer line temperature was 225°C. A solvent delay of 300 seconds was used. The ion source temperature was 250°C with an electron impact (EI) energy of 70 eV. The collected mass range was 35–600 amu. The acquisition rate was 100 scans per second and the detector voltage was 1500 V.

### 5. Data Processing

Data processing and display of the GCxGC chromatograms were achieved using the integrated LECO ChromaTOF™ software, version 4.33. Peak apexes of deconvoluted signals were found automatically and were further corrected manually when required. The ^2^D chromatographic peaks were recombined based on their second dimension retention time (^2^t_R_) and mass spectral similarity. Mass spectral data (DIC) were compared to the Wiley (2008) and the NIST (2008) libraries for temptative identification (similarity >700 and reverse match >900). Moreover, the area of each peak was calculated based on the apex mass reconstruction trace to increase the specificity during the comparison. All sample-blank couples were analyzed using the “Reference” option of the software to compare every sample to its blank. This part of the software is based on an algorithmic comparison of the data sets [51]. To analyze one sample-blank couple, the comparison is based on both ^1^t_R_ and ^2^t_R_, as well as the respective area and mass spectra data of each peak. The main comparison parameters were fixed at a tolerable shift of 8 seconds on ^1^t_R_ (twice P_M_ value) and 0.2 seconds on ^2^t_R_, and a tolerance of 50% absolute variation on the area. Following this comparison exercise, every single peak was categorized in one of the following groups: match (the compound is found in both injections), not found (the compound is only present in the sample), out of tolerance (the compound is present in both injections but at a different concentration level), and unknown (the compound is only present in the blank). The compounds sorted in the “not found” and “out of tolerance” (with a concentration under 10%) groups were classified as specific to the decomposition process.

### 6. Decomposition Stages

The decomposition process was observed according to different stages. We decided to discriminate five major decompositional stages based on different visual criteria adapted from the literature [50], [52]–[58]: (1) fresh, (2) bloated, (3) active decay, (4) advanced decay and (5) dry remains. The decay stages are presented in Table 1.

### 7. Statistical Analysis

In order to analyze the spatial distribution of our data set, a statistical analysis was conducted through multivariate principal component analysis (PCA) (Minitab® v15.1, State College, PA, USA). The original data set is a matrix of c×n (c  =  objects, n  =  variables) corresponding to a matrix of 12×225. Chemical compounds emitted only once were excluded from the multivariate analysis. The relative area of the chromatogram's peak corresponding to VOC was used for the multivariate analysis.

## Results

### 1. Environmental Parameters

The mean atmospheric temperature measured during the decay process was 13.1°C. Figure 1 shows the temperature recordings in the forest site. The mean atmospheric maximal temperature was 20.2°C whereas the mean atmospheric minimal temperature was 6.7°C. The mean relative humidity was 68.3%. Local atmospheric temperature and relative humidity values were different to seasonal averages reported for the last 20 years by the royal Belgian meteorological institute (KMI-IRM). Mean atmospheric temperature from the Belgian KMI-IRM archives for spring 2007 (March to May) was 12.3°C (14.3°C for April and 14.6 for May 2007) whereas seasonal average was 9.5°C (9°C for April and 12.7°c for May). The maximal mean temperature from the Belgian KMI-IRM archives, for April 2007, was 20.5°C and 19°C for May. The minimal mean temperature from the Belgian KMI-IRM archives was 7.6°C for April 2007 and 10.3°C for May. The seasonal average for maximal temperature was 13.1°C for April and 17.2°C for May. For minimal temperature, the seasonal average was 5°C for April and 8.3°C for May. Spring 2007 was the warmest season since 100 years. Mean relative humidity from the Belgian KMI-IRM archives was 62% for April 2007 and 75% for May whereas seasonal averages were respectively 76.6% and 75.5%. Moreover, there was no day of precipitations during April 2007.

### 2. Decay Stages

Figure 2 illustrates the decay stages followed by the swine carcass in the forest biotope. The “fresh” stage began the day of the death (March 29, 2007) until 2^nd^ of April, *i.e.* five postmortem days. The “bloating” stage began on the 3^rd^ of April and finished on the 17^th^ of April, the duration of the bloating stage was fifteen days. The “active decay” stage began on the 18^th^ of April until the 30^th^ of April. The duration of the active decay stage was thirteen days. The “advanced decay” stage began on the 1^st^ of May until the 11^th^ of May; this decomposition stage had duration of eleven days. The decay process was followed during six postmortem weeks.

### 3. Two-dimensional Chromatographic Screening

Figure 3 shows the GCxGC apex plot of a sample (1^st^ of May) of the advanced decay stage. Figure 3A clearly illustrates the added value of GCxGC for such analyses for which classical GC would suffer from repeated co-elution issues (same ^1^t_R_). After processing, the peak table of this chromatogram contained 633 hits. Hits included in the circled region could easily and repeatably be attributed to the extraction solvent. Hits included in the rectangle region were related to column bleed. After further removal of the hits present in the reference samples, 218 peaks were isolated (Figure 3B). Amongst these peaks, 42 were specifically found with at least 4 occurrences in other sample extracts (e.g. part of the 60 compounds listed in Table 2, see next paragraph). Figure 3C illustrates their distribution over the chromatographic space. All samples were processed the same way before compilation of the list of specific compounds.

### 4. Cadaveric Volatile Organic Compounds

More than 4,000 hits were reported from extract analyses. After clean-up of the lists from compounds present in reference soils, as well as from GC column bleed related and potential peak artifacts, a list of 830 VOCs specifically released under the pig decomposition process was extracted by GCxGC-TOFMS. Almost all chemical families of VOCs were represented (in parentheses, the number of chemical compounds identified): alkanes (*i.e.* saturated hydrocarbons) (82), alkenes (*i.e*. unsaturated hydrocarbons) (50), alcohols (64), carboxylic acids (45), aromatic compounds (84), esters (87), sulfur compounds (31), nitrogen compounds (141), aldehydes (32), ketones (110), halogen compounds (42), ethers (32) and unclassed compounds (30). Table 3 presents the 225 cadaveric VOCs which have at least two occurrences during the decay process; 605 chemical compounds were detected only once. Among these 225 chemical compounds, 1H-indole was the compound with the highest occurrence (eight occurrences), followed by five compounds with seven occurrences (2-methyl-1-pentene; 1,2,3-propanetriol; ethanol; 4-methylphenol and acetaldehyde). Ten compounds were found 6 times, for example: 1-butanol, butanoic acid, 2-methylpropanoic acid, DMDS (*i.e*. dimethyldisulfide) and DMTS (*i.e.* dimethyltrisulfide), 1-amino-2-propanol, N-butylformamide and N,N-formamide. Sixteen compounds had five occurrences, for instance: 2- and 3-methylbutanoic acid, pentanoic acid, trimethylamine, 2-octanone and 2-undecanone, nonanal. An exhaustive list of compound occurrences (only the chemical volatiles with four to eight occurrences) is compiled in Table 2. Figure 4 shows the number of cadaveric volatile chemicals released by sampling date. The highest number of postmortem compounds was monitored during the decay stages, more precisely during the active decay stage and early advanced decay stage. The active decay is the decompositional stage with the strongest olfactive signature as many chemicals were detected. On the contrary, few volatiles were detected during the fresh stage (one day postmortem time). Additionally, no decompositional odor was perceptible to the human sense of smell in the very early decompositional stages. The number of cadaveric VOCs increased with the course of time and decreased with the disappearance of soft tissues (advanced decay). The PCA diagram (Figure 5) shows the distribution of the postmortem time (dates) in a score-plot (F1, σ = 57.501; F2, σ = 26.086). The fresh and bloated stages are less differentiated from the decay stages. Figure 6 shows the repartition of chemical families detected in the headspace of decaying swine carcass by postmortem time. The volatile pattern of decaying pig carcass changes over time. One day post-mortem (30^th^ of March), alkene was the predominant chemical class with γ-terpinene and o-xylene. In the fresh decay stage (5 postmortem days), alcohol was the predominant chemical class with mainly 1-butanol. 8 postmortem days, the presence of alcohols decreased and ketones became the main chemical class with principally cyclohexanone (≈ 20% of the total of emitted volatiles) and 4-methyl-3-pent-2-one. Aldehydes (hexanal, pentadecanal, 2,2-dimethylpropanal, 3-methylbutanal, acetaldehyde) were also more abundant and represented approximately 15% of the total of emitted volatiles. Furthermore, in bloated stage, alcohols represent more than a third of the volatile emissions whereas carboxylic acids represented 35% with mainly one chemical compound (borinic acid, diethyl-). Nitrogen compounds became more important and represented moreover 10% of the total of volatile emissions. 15 postmortem days, aldehydes, aromatic and sulfur compounds were the main chemical classes. 2,2 dimethylpropanal was the predominant aldehyde and represented a third of the total of volatile emissions whereas 1H-indole (aromatic compound) represented approximately a quarter of the volatile emissions. With regards to sulfur compounds, dimethyltrisulfide (*i.e.* DMTS) and butyl isopropylsulfone were the predominant compounds detected. During the active decay stage, nitrogen compounds (1-butanol, 4-amino; trimethylamine) and sulfur compounds (DMTS ≈ 23% of the total of volatile emissions of 22 postmortem days) are the main chemical families. Butanoic acid (carboxylic acid) became more important in the volatile emissions. 26 postmortem days, 75% of the volatile emissions were carboxylic acids with 3-methylbutanoic acid (*i.e*. isovaleric acid), butanoic acid, 2-methylbutanoic acid, 2-methylpropanoic acid (*i.e.* isobutyric acid), 4-methylpentanoic acid (*i.e.* isocaproic acid). Many aromatic compounds (37 chemical compounds) were also detected on the 26 postmortem days, but the main compound was 4-methylphenol (*i.e. p*-cresol). 29 postmortem days, the main chemical class was still the carboxylic acids (butanoic acid, 2- and 3-methylbutanoic acid, 2-methylpropanoic acid) with moreover 80% of the total of volatile emissions. In the early advanced decay stage (33 to 36 postmortem days), the presence of carboxylic acids decreased and the predominant chemical classes were ketones and alkanes. Further in the advanced decay stage, the alkanes increased; the main saturated hydrocarbon was 1,3-diethylcyclopentane (≈50% of the total of volatile emissions of 36 postmortem days). Carboxylic acids, approximately 20% of the total of volatile emissions, were still present but to a lesser extent. 40 postmortem days, sulfur compounds were the predominant chemical class with 4-hydroxybenzenesulfonic acid and dimethyldisulfide (DMDS), which represented more than 60% of the volatile emissions. Aldehydes (mainly nonanal) and aromatic compounds (mainly 1H-indole) intervened respectively for 9% and 14% of the total of volatile emissions. Later in the advanced decay stage (43 postmortem days)), sulfur compounds (main compound: DMDS) decreased whereas ketones (2-hexanone, 3-hexanone and cyclohexanone), nitrogen compounds and carboxylic acid increased (2,3-dihydroxysuccinic acid).

## Discussion

In previous study on swine decay chemistry (above ground decomposition in a forest biotope) [14], only 85 specific cadaveric volatiles were identified with conventional GC-MS whereas approximately ten times more compounds were detected with two-dimensional GCxGC-TOFMS (832 VOCs). Moreover, no cadaveric compounds were detected with conventional GC-quadrupole(q)MS during the first postmortem week (fresh decay and beginning of the bloated stages) [14] whereas cadaveric volatiles were detected from the first postmortem day with bi-dimensional gas chromatography. This is most probably to be attributed to the enhanced chromatographic resolution and detectability of the zone-compressed peaks. Figure 3A also illustrates that separating column bleed and/or solvent related peaks in the second dimension contributes to better peak identification. Furthermore, the use of the chromatographic space rather than a classical chromatographic temporal line allows classes of compounds to be separated from each other (Figure 3C). Amides, aromatics, carboxylic acids exhibit different retention behaviors towards the ^2^D GC phase. The availability of two t_R_ values is an additional piece of information to potentially identify compounds with lower similarity values versus library spectra. The separation could still be improved for alcohols, ketones, carboxylic acids, and aldehydes, but the current situation is clearly improved compared to a situation where all peaks would be present on the top of each other on the x-axis of the chromatogram. For now, the deconvolution software of the TOFMS system can separate them for individual identification.

Other studies on the swine cadaveric decompositional process [7], [14] or human decay [7]–[12] used GC-qMS [7]–[13], [15], [17] or GC-TOFMS [7] to analyze the decompositional odor. However, fewer cadaveric compounds were detected with GC-MS or GC-TOFMS than the two-dimensional gas chromatography. For example, in the Decompositional Odor Analysis Database (DOA Database), 478 specific volatile compounds associated with buried human remains were recovered [8]–[9] whereas Statheropoulos and colleagues [3] detected more than 80 VOCs on putrefied human remains and 150 VOCs were identified on pig carcasses closed in a “body bag” during the early stages of decay [7]. The volatile profiles of different types of human remain (blood, bones, adipose, teeth, skin, body fat, *etc*.), placed in small sealed vials, were constituted of 33 specific VOCs [12].

A recent study [16] used the two dimensional GCxGC-TOFMS analysis to study the chemical composition of volatiles emanating from fresh laboratory mouse carcasses during the early decay stages. A fresh killed mouse (0–30 min old) emits the same volatile pattern as a living mouse. As the decay progresses, only sulfur compounds (S-VOCs) were detected during the first three days of postmortem time. In older mice carcasses, other decompositional by-products were identified such as aromatic phenolic compounds (indole, scatole), amines and mercaptoacetic acid [16]. However, the olfactive signature of decaying mouse carcasses was not completely described and, except for S-VOCs, few cadaveric compounds were referenced in Kalinova and colleagues’ [16] study. Again, their volatile samplings on mouse models were made in a small closed environment with solid-phase microextraction (SPME) during the beginning of the decompositional process, which may explain the differences. Indeed, postmortem volatiles may be concentrated in closed environments and are detectable earlier.

There is a large discrepancy in decomposition odor compounds reported in the literature [13]. This difference could be explained by the various analytical protocols, including volatile extractions (sampling techniques and sorbent materials) and analytical separations, used to study the postmortem volatiles of vertebrate tissues [4], [59]. Moreover, the result of volatile analysis from decomposition of mammalian soft tissues is also influenced by abiotic factors such as temperature and moisture [4]. The use of different mammalian models or remains (tissues samples *vs.* whole corpses) could also lead to differences in cadaveric volatile profiles. There are some recommendations to examine the potential differences between human and animal models [25]. Indeed, the olfactive signature of decaying pig carcasses shows similarities with the smell of human decomposition in terms of released chemical compounds [13]–[14], but also dissimilarities [13]. Recent research in this field indicated that the odor from human remains is different from that of animals [13]. Two compounds were exclusively found on human remains and not on animal samples: styrene and benzoic acid methyl ester [13]. These two compounds were not detected in our pig decaying samples. However, the principal identified cadaveric VOCs are, in general, in agreement with previous studies conducted on pig decaying carcasses [7], [14]–[15]. These cadaveric compounds come from the chemical breakdown of the principal body constituents: proteins, lipids, nucleic acid and carbohydrates [2]–[3], [25]. Indole and other phenolic compounds (*e.g*. *p*-cresol) might originate from protein and fat decomposition [1], [7]. Indeed, proteolysis (*i.e.* the breakdown of proteins by the action of bacterial enzymes [5], [60]) yields gases, diamines (cadaverine, putrescine), sulfur compounds and phenolic compounds including indole and skatole [1], [3], [5], [60]–[61].

Skatole (*i.e.* 3-methylindole) was not detected in the present study, confirming the findings of the previous study [14]. In addition, cadaverine and putrescine were not detected as cadaveric VOCs in this study. The absence of these biogenic amines, usually associated with the decay process [2], [49], is also confirmed in previous studies on volatile chemistry of human decay [3], [8]–[9] or swine decay [7], [14]. Indeed, biogenic amines have a low volatility and therefore are not frequently identified with gas chromatography [4]. Liquid chromatography (LC) is more suitable to detect biogenic amines [4]. Even so, 2-piperidone, a cadaverine metabolite [62], was detected in our cadaveric emanation. Sulfur compounds such as DMDS and DMTS are very frequent biomarkers in cadaveric samples of vertebrate tissues [3]–[5], [7]–[17]. Volatile sulfur compounds (VSCs or S-VOCs) come from the microbial breakdown of sulfur-containing amino acids (cysteine, methionine) [7]. The oxygenated compounds (organic acids, alcohols [3], [5], [49], ketones, aldehydes, esters and ethers [3]) come from the carbohydrate decomposition. The breakdown of nucleic acids provides nitrogenous bases, phosphates and sugars [2] whereas lipid degradation produces mainly fatty acids, hydrocarbons, oxygenated, phosphorus and nitrogen compounds [3], [49]. Nevertheless, it is important to remember that for some cadaveric compounds the pathways of their formation are not known in detail or are still completely unknown [4], [7].

It is interesting to note that the dothiepin (also called dosulepin [63]: 3-(dibenzo[b,e]thiepin-11(6H)-ylidene)-N,Ndimethylpropylamine [64]–[65]) was frequently found in our postmortem volatile samples. However, dothiepin is not a specific cadaveric VOC. Dothiepin is a tricyclic antidepressant with tranquilizing properties [63]–[65]. This compound was probably dosed antemortem to the pig by the veterinarian to improve the animal welfare. Indeed, psychotherapeutic drugs such as tricyclic antidepressants can be used to eliminate the anxiety-related behavior of animals [66]. To the best of our knowledge, this is the first time that drug was detected on a decaying corpse with headspace collection and bi-dimensional gas chromatography. Nevertheless, some basic drugs, including dothiepin, could be detected in postmortem blood samples using gas chromatography coupled to mass spectrometry with ion trap detection [67]. The headspace detection of volatile compounds of forensic interest (*e.g.* drugs, ethanol) on decaying corpse opens up new possibilities in forensic toxicology. Recent research has been conducted into the development of direct headspace sampling methods to analyze volatile compounds of forensic interest in human biological fluids [68]–[69].

In conclusion, this study provides the first documentation of the use of GCxGC-TOFMS to analyze pig decaying volatile compounds. The use of comprehensive GC could improve the characterization of the smell of death in terms of volatile constitution, rather than conventional GC. Indeed, the complexity of postmortem volatile samples requires more complex analytical methods [25]. Concerning data analysis, it would be interesting to include chemometrics analysis in future work. Nevertheless, the solvent extraction of the volatile organic compounds from the sorbent cartridges as well as the storage of the liquid fraction prior analyses is not adequate for the most volatile polar compounds, compared to the use of thermal desorption techniques, which is currently under investigation. However, our results demonstrated that bi-dimensional gas chromatography coupled with time-of-flight mass spectrometry is a powerful tool to analyze the volatile cadaveric emissions.
